# Profiling genetic variants in cardiovascular disease genes among a Heterogeneous cohort of Mendelian conditions patients and electronic health records

**DOI:** 10.3389/fmolb.2024.1451457

**Published:** 2024-10-01

**Authors:** Nadia Akawi, Ghadeera Al Mansoori, Anwar Al Zaabi, Andrea Badics, Noura Al Dhaheri, Aisha Al Shamsi, Amal Al Tenaiji, Bashar Alzohily, Fatmah S. A. Almesmari, Hamad Al Hammadi, Nahid Al Dhahouri, Manal Irshaid, Praseetha Kizhakkedath, Fatema Al Shibli, Mohammed Tabouni, Mushal Allam, Ibrahim Baydoun, Hiba Alblooshi, Bassam R. Ali, Roger S. Foo, Fatma Al Jasmi

**Affiliations:** ^1^ Department of Genetics and Genomics, College of Medicine and Health Sciences, United Arab Emirates University, Al Ain, United Arab Emirates; ^2^ Division of Cardiovascular Medicine, University of Oxford, Oxford, United Kingdom; ^3^ Department of Cardiology, Sheikh Shakhbout Medical City, Abu Dhabi, United Arab Emirates; ^4^ Department of Cardiology, Tawam Hospital, Al Ain, United Arab Emirates; ^5^ Genetic Metabolic Division, Pediatrics Department, Tawam Hospital, Al Ain, United Arab Emirates; ^6^ Department of Pediatrics, Sheikh Khalifa Medical City, Abu Dhabi, United Arab Emirates; ^7^ Antimicrobial Research Unit, School of Health Sciences, University of KwaZulu-Natal, Durban, South Africa; ^8^ Cardiovascular Research Institute, Centre for Translational Medicine, National University Health System, Singapore, Singapore; ^9^ Genome Institute of Singapore, and Institute of Cardiovascular Sciences, University of Birmingham, Birmingham, United Kingdom

**Keywords:** heritable cardiovascular disease, variants, genes, signalling pathways, mendelian study cohort, electronic health records, United Arab Emirates population

## Abstract

**Introduction:**

This study addresses the rising cardiovascular disease (CVD) rates in the United Arab Emirates (UAE) by investigating the occurrence and impact of genetic variants in CVD-related genes.

**Methods:**

We collected all genes linked to heritable CVD from public and diagnostic databases and mapped them to their corresponding biological processes and molecular pathways. We then evaluated the types and burden of genetic variants within these genes in 343 individuals from the Emirati Mendelian Study Cohort and 3,007 national electronic health records.

**Results:**

We identified a total of 735 genes associated with heritable CVD, covering a range of cardiovascular conditions. Enrichment analysis revealed key biological processes and pathways, including Apelin, FoxO, and Ras signaling, that are implicated across all forms of heritable CVD. Analysis of a UAE cohort of 3,350 individuals showed a predominance of rare and unique CVD variants specific to the population. The study found a significant burden of pathogenic variants in families with CVD within the Emirati Mendelian cohort and re-assessed the pathogenicity of 693 variants from national health records, leading to the discovery of new CVD-causing variants.

**Discussion:**

This study underscores the importance of continuously updating our understanding of genes and pathways related to CVD. It also highlights the significant underrepresentation of the UAE population in public databases and clinical literature on CVD genetics, offering valuable insights that can inform future research and intervention strategies.

## Graphical Abstract

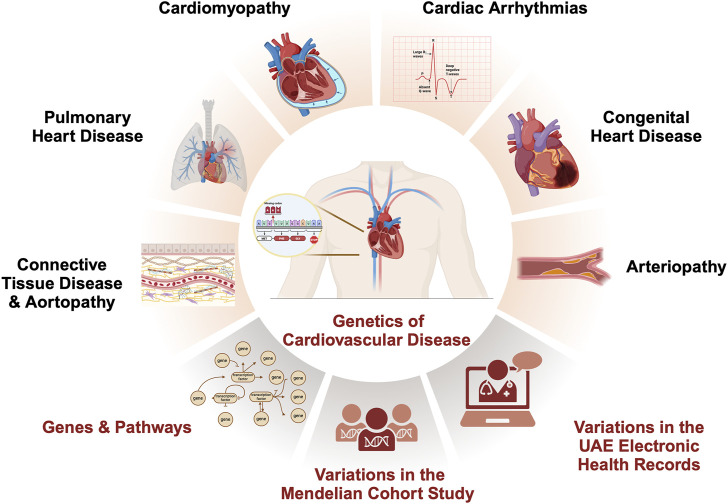

## Introduction

Due to rapid economic expansion and influx of immigrants, the United Arab Emirates (UAE) is transforming in various domains. This is mainly associated with the prevalent adoption of unhealthy lifestyles among the population, significantly impacting individual health. One of the imminent health issues in the country is the rising rate of cardiovascular disease (CVD)-related morbidity and mortality. In UAE, more than 40% of all deaths are attributable to CVD, with 39.7% in the emirate of Abu Dhabi (Ghader et al., 2023; STATISTICS, 2017). The findings from a 9-year longitudinal study conducted in the UAE showed that the 9-year cumulative incidence of major CVD was 9.9%, surpassing its cumulative incidence among Europeans, estimated to be around 4.7% (Al-Shamsi et al., 2019; Al-Shamsi, 2020). To combat this public health crisis, we need to understand the root causes of CVD in this population to improve primary prevention and treatment of CVD.

**FIGURE 1:**
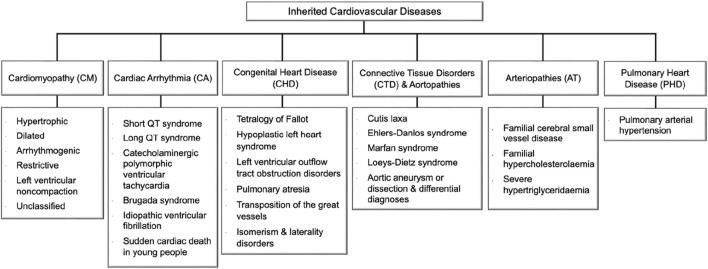
Inherited cardiovascular disease main categories and relevant disorders that meet rare disease eligibility criteria accepted by the experts’ community of the National Health Service (NHS) in England (https://panelapp.genomicsengland.co.uk/panels/). Cardiomyopathies (CM) are heart muscle diseases that can lead to abnormal heart function and, ultimately, heart failure. The most common types are hypertrophic cardiomyopathy and dilated cardiomyopathy. Cardiac arrhythmias (CA) are abnormalities in the rhythm or rate of the heartbeat. Some cardiac arrhythmias are known to have a genetic component, including Long QT syndrome and Brugada syndrome. Congenital heart disease (CHD) encompasses a range of structural abnormalities in the heart that are apparent from birth, including atrial and ventricular septal defects. Connective tissue disorders (CTD) are a group of disorders that affect the tissues that support and connect various structures in the body, primarily the heart and blood vessels. Many CTDs can lead to weakening or dilatation of the aorta, which can cause life-threatening complications such as aortic aneurysms and dissections collectively named aortopathies such as Marfan syndrome, Loeys-Dietz syndrome, and vascular Ehlers-Danlos syndrome. Inherited arteriopathies (AT) encompass familial hypercholesterolemia and hypertriglyceridemia, genetic disorders that cause very high levels of LDL cholesterol and triglycerides in the blood. Pulmonary heart disease (PHD) is a condition that occurs when the pulmonary arteries, which carry blood from the heart to the lungs, become narrowed or blocked, causing increased pressure in the heart and leading to heart failure.

Encompassing a broad spectrum of conditions, the majority of CVDs have a hereditary basis due to genetic variations that either cause or increase the risk of developing the disease (Figure Figure1) (Vrablik et al., 2021). Understanding the genes and genetic variants responsible for the heritability of CVD is a crucial aspect of the UAE’s current research agenda to match international efforts while considering local needs (Ghader et al., 2023). Considering all genes associated with heritable CVD (Williams et al., 2019; Maron et al., 2018; Goyal et al., 2017; Schwartz et al., 2020; Austin and Loyd, 2014; Hu et al., 2020), we aim in this study to identify CVD variants detected in the UAE by retrospectively analyzing genetic testing data from 343 individuals in a local cohort study and 3,007 individuals from the national electronic health records (Supplementary Figure S1).

## Methods

The study design is illustrated in Supplementary Figure S1.

### Preparation of heritable cardiovascular diseases gene list

The list of CVD disorders that met the rare disease eligibility criteria and associated genes was obtained mainly from Genomics England Panels (https://panelapp.genomicsengland.co.uk/panels/), developed by the 100K Genomes project. We also included genes from diagnostic panels, including CENTOGENE (https://www.centogene.com), Blueprint Genetics (https://blueprintgenetics.com), and CeGat (https://cegat.com). Followed by manual curation through a literature survey in the PubMed electronic databases using five MeSH terms, namely, cardiomyopathy (CM), cardiac arrhythmia (CA), congenital heart disease (CHD), connective tissue disease (CTD), arteriopathies (AT), and pulmonary heart disease (PHD). Five lists of genes were generated for all disease categories.

The list of heritable CVD actionable genes was retrieved from the latest ACMG Recommendations for Reporting of Secondary Findings in Clinical Exome and Genome Sequencing via NCBI (https://www.ncbi.nlm.nih.gov/clinvar/docs/acmg/).

### The Emirati Mendelian Study cohort and patients

Patient blood samples were sent to the UAE University Genomic Laboratory at the College of Medicine and Health Sciences (Al Ain, UAE) between July 2020 and May 2021. The patients or their relatives provided written informed consent for genetic investigation and research following the Declaration of Helsinki and French Law. The Institutional Review Board approved this study at the Department of Health (Abu Dhabi, UAE; DOH/CVDC/2020/1185). The baseline characteristics of the Mendelian study participants are shown in Supplementary Table S1.

### Exome sequencing

Genomic DNA extraction from peripheral blood was performed using the QIAamp DNA Blood Mini Kit (Qiagen, Germany) on the QIAcube instrument (Invitrogen, USA). The DNA’s quality was assessed using a Nanodrop One Spectrophotometer (Thermo Fisher Scientific, USA) and an Agilent 4,200 TapeStation system (Genomic DNA ScreenTape Assay; Agilent Technologies, USA), while the quantity was measured using a Qubit 4.0 Fluorometer (Qubit dsDNA BR Assay kit; Invitrogen, USA).

Exome sequencing (ES) was conducted at the United Arab Emirates University (UAEU) Genomics Laboratory. ES library preparation was carried out using the TruSeq Exome Enrichment kit (Illumina, USA) following the manufacturer’s protocol. Briefly, genomic DNA was fragmented using LE220-plus Focused-ultrasonicator (Covaris, USA) to generate 150 bp insert libraries. The DNA fragments of each sample were end-repaired and ligated to dual-index adaptors. A Qubit 4.0 Fluorometer (Qubit dsDNA HS Assay kit; Invitrogen, USA) and an Agilent 4,200 TapeStation system (D1000 and HS D1000 ScreenTape Assays; Agilent Technologies, USA) were used to determine the libraries’ concentrations and fragment size Prior sequencing, The final quantified libraries were pooled, normalized, and then sequenced with paired-end reads (2 × 150 bp) on the NovaSeq 6,000 System (Illumina, USA) employing S2 flow cell. A combination of in-house developed pipelines and the Illumina DRAGEN Bio-IT Platform (Illumina, USA) was used for read mapping, alignment, variants calling, and quality checks.

### Genomic annotation and analysis

Gene annotation was performed using VarSeq 2.2.4 software (Golden Helix, USA). Gene ontology enrichment analysis was conducted in the Gene Ontology (GO) knowledgebase. Pathway enrichment analysis was performed using ConsensusPathDB (http://cpdb.molgen.mpg.de). Ensembl Variant Effect Predictor (VEP; https://asia.ensembl.org/info/docs/tools/vep/index.html) and VarSeq 2.2.4 software (Golden Helix, USA) were used for variants’ annotation and filtration. Minor allele frequencies (MAFs) of all variants were retrieved from the gnomAD database (http://gnomad.broadinstitute.org). Variants’ pathogenicity was classified according to the American College of Medical Genetics and Genomics (ACMG) classification framework (Richards et al., 2015) and patients’ phenotypes. We used Fisher’s exact test to assess the enrichment of a particular class of CVD variation in patients.

### Retrieving cardiovascular disease-related variants from the electronic health records

We reviewed the Abu Dhabi Health Company SEHA Electronic Health Record (EHR) of patients accessing care between 2011 and June 2019 (8 years) in Tawam Hospital, a tertiary hospital in Al Ain, and the referral genetic center in the UAE. This retrospective review of EHR was approved by the SEHA Research Ethics Committee (SEHA-IRB-028). We identified more than 26,275 records of individuals who underwent genetic testing, including chromosomal microarray and sequencing. We extracted 4,860 laboratory reports of genetic variation data for 3,007 individuals. Next, we filtered these records for variants falling in our set of CVD genes. We annotated these variants using Franklin (https://franklin.genoox.com).

## Results

### Aggregating cardiovascular disease genes dataset and revealing their role in cardiovascular biology

**FIGURE 2:**
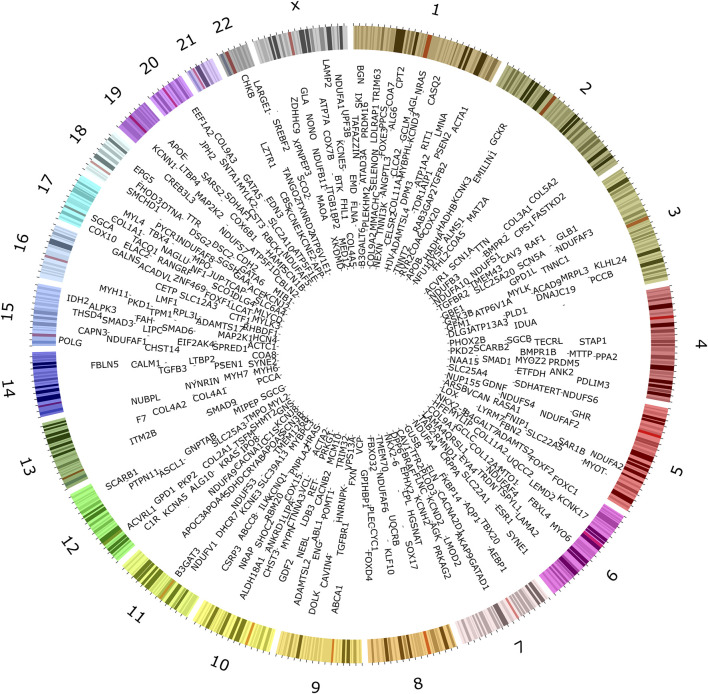
Genes associated with inherited forms of cardiovascular disease. Extensive searches in public databases, diagnostic panels, and related literature revealed 735 genes associated with specific heritable CVD categories.

To understand the current landscape of CVD genetics, we compiled a list of CVD-associated genes from clinical databases, identifying 735 genes (Figure 2; Supplementary Table S1). We classified the identified CVD gene set based on their disease categories (Supplementary Figures S2-S7). Among these categorizations, 298 genes (32%) were linked to CM (Supplementary Figure S2), 160 genes (18%) were associated with CA (Supplementary Figure S3), 275 genes (30%) were identified with CHD (Supplementary Figure S4), 93 genes (10%) were linked to CTD (Supplementary Figure S5), 64 genes (7%) were found associated with AT (Supplementary Figure S6), and 31 genes (3%) were connected to PHD (Supplementary Figure S7).

We analyzed gene ontology enrichment (GO) to explore the CVD genes’ functional characteristics (Supplementary Table S8). A more than 20-fold enrichment in GO terms related to muscle structure and function, cardiac development and function, left/right asymmetry and organ development, cell communication and signaling membrane function, lipoprotein and lipid metabolism, cell migration and morphogenesis, and regulation of cellular processes (Supplementary Figure S8).

**FIGURE 3:**
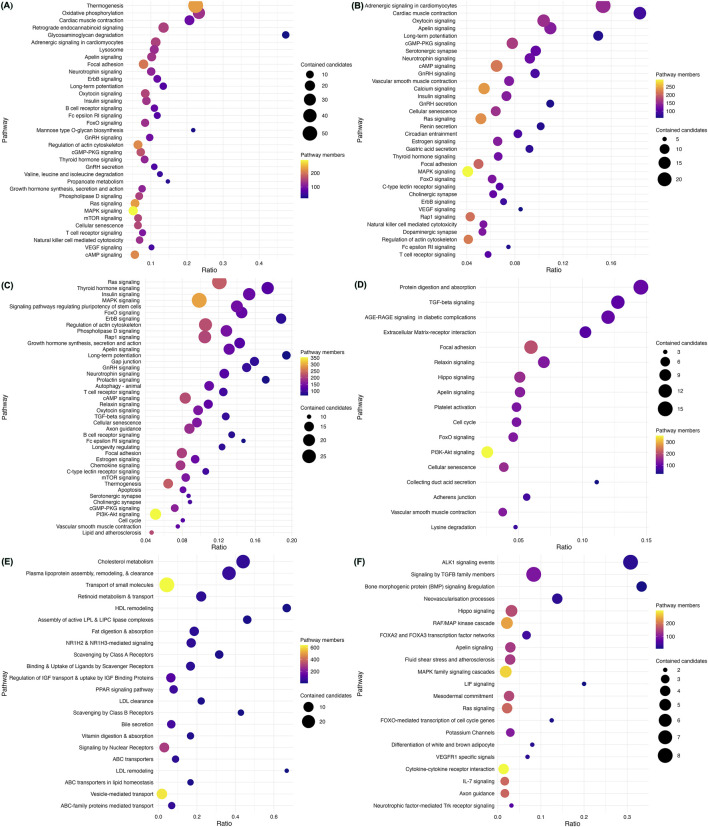
Enriched pathways in heritable cardiovascular diseases. Pathway enrichment analysis of the 735 CVD-associated genes using ConsensusPathDB revealed mechanisms underlying each category. Cardiomyopathy genes were found to be significantly enriched in non-shivering thermogenesis (NST) and mitochondrial oxidative phosphorylation (mOXPHOS) **(A)**. Adrenergic signaling and cardiac muscle contraction were the top-ranked pathways enriched with CA genes **(B)**. CHD genes were enriched in key signaling pathways such as Ras, Thyroid, Insulin, and MAPK **(C)**. CTD genes were located primarily in extracellular matrix (ECM) organization **(D)**. AT genes were enriched in lipid metabolism and homeostasis **(E)**. PHD genes were enriched in ALK1 and TGFB signaling **(F)**. The scatter plots display representative sets of enriched pathways across all forms of CVD at an FDR <0.001 and usually contain more than five genes, sorted by their significance (q-value). Pathway names are depicted on the vertical axis, while the horizontal axis indicates the enrichment ratio (number of hits/total). Each data point’s color corresponds to the pathway’s size, and the size reflects the number of CVD genes associated with that pathway.

We assessed the enrichment of CVD gene sets from each disease category within mechanisms potentially contributing to the pathogenesis of CVD (Figure 3; Supplementary Tables S3-S8). CM genes were significantly enriched in nearly 82 pathways at an FDR <0.003 (Supplementary Table S3). Out of these, non-shivering thermogenesis (NST) and mitochondrial oxidative phosphorylation (mOXPHOS) were the top pathways (Figure 3A). Out of 93 pathways enriched with CA genes, adrenergic signaling and cardiac muscle contraction were top-ranked hits (Figure 3B; Supplementary Table S4). CHD genes were enriched in 65 pathways, with top hits being the critical signaling pathways Ras, Thyroid, Insulin, and MAPK (Figure 3C; Supplementary Table S5). Out of 42 pathways, CTD genes were enriched primarily in extracellular matrix (ECM) organization (Figure 3D; Supplementary Table S6). AT genes were enriched in 35 pathways, with the top hits being lipid metabolism and homeostasis (Figure 3E; Supplementary Table S7). PHD genes were enriched in 26 pathways, of which ALK1 and TGFB signaling were the top hits (Figure 3F; Supplementary Table S8).

We noted a substantial overlap between genes associated with CVD heritable forms, especially between CM and CA (Supplementary Table S9; Supplementary Figure S9). More than 19.4% (89/458) of genes were shared between CM and CA, mapping to 23 pathways indicating a common pathogenesis (Supplementary Figures S9 and S10). These include cell signaling pathways (such as neurotrophin signaling, VEGF signaling, ErbB signaling, FcεRI signaling, insulin signaling, oxytocin signaling, apelin signaling, adrenergic signaling in cardiomyocytes, MAPK signaling, GnRH secretion, cAMP signaling, thyroid hormone signaling, FoxO signaling, T-cell receptor signaling, Ras signaling, GnRH signaling, cGMP-PKG signaling), and cellular processes (such as cardiac muscle contraction, regulation of actin cytoskeleton, long-term potentiation, cellular senescence, focal adhesion, natural killer cell-mediated cytotoxicity).

### Evaluating the occurrence and impact of cardiovascular disease genes and variants in the Emirati Mendelian Study cohort

To understand the genetics architecture of CVD in the UAE population, we investigated CVD-associated genes and variants from the Emirati Mendelian study dataset encompassing exomes of 343 individuals. The cohort includes 134 families, and 115 patients diagnosed with rare Mendelian disorders were recruited from various clinics in seven UAE hospitals (Table 1). The patients exhibited diverse phenotypic manifestations, encompassing physical abnormalities that impacted their growth, skeletal structure, vision, hearing, neurological function, cardiac health, pulmonary, renal, and liver function.

**TABLE 1:** Baseline characteristics of sequenced participants in the Emirati Mendelian study.

Characteristic	Number	%
Cohort	**343**	**100.00**
Families	134	39.10
Patients	115	33.50
Age, y	**309**	**90.10**
Children (<2 and >18)	140	45.30
Adults (≥18)	169	54.70
Sex	**338**	**98.50**
Male	159	47.00
Female	179	53.00
Referral Clinic	**343**	**100.00**
Cardiovascular Clinic	46	13.40
Genetics Clinic	172	50.10
Endocrinology Clinic	3	0.90
Ear, Nose & Throat Clinic	39	11.40
Nephrology Clinic	50	14.60
Ophthalmology Clinic	20	5.80
Rheumatology Clinic	13	3.80
Country of Origin	**311**	**90.70**
UAE	84	27.00
Pakistan	44	14.10
Egypt	35	11.30
Jordan	31	10.00
Syria	28	9.00
India	14	4.50
Yemen	14	4.50
Afghanistan	13	4.20
Palestine	8	2.60
Iraq	6	1.90
Oman	6	1.90
Comoros	5	1.60
Sudan	5	1.60
Bangladesh	3	1.00
Iran	3	1.00
Mauritania	3	1.00
Somalia	3	1.00
Philippines	2	0.60
Saudi Arabia	2	0.60
Morocco	1	0.30
Nepal	1	0.30

Bold font indicates a characteristic headline.

From this cohort, we retrieved all variants in our CVD gene set (Supplementary Table S10). We were able to obtain 23,097 variants falling within 643 CVD genes. Most of these genes cause multiple phenotypes transmitted in different modes of inheritance (Supplementary Table S10). We extracted reported variants in HGMD for each gene (Supplementary Table S10). Thousands of HGMD damaging variants were clustered in *NF1*, *FBN1*, *DMD*, *PKD1*, *LDLR*, *SCN1A*, *COL1A1*, *ATM*, *COL4A5*, *TTN* genes.

**FIGURE 4:**
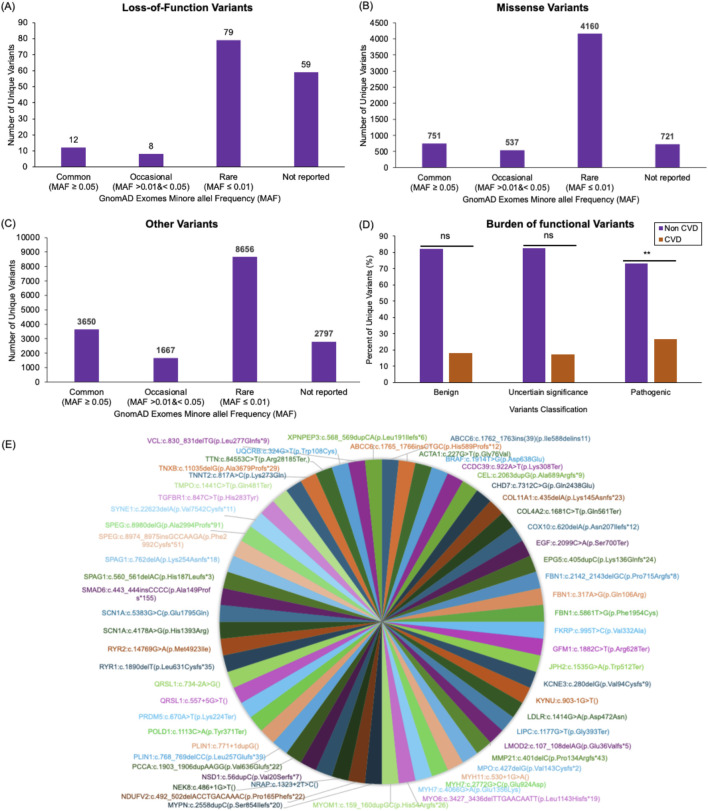
Cardiovascular disease variants profile in the Mendelian study cohort. Bar plots display the number of unique loss-of-function **(A)**, missense **(B)**, and all other **(C)** variants found within genes associated with CVD in the 343 individuals analyzed here. These variants are categorized based on their minor allele frequency (MAF) in the gnomAD database. Variants reported in gnomAD with a MAF ≥0.05 were classified as Common variants. Reported variants with MAF >0.01 and <0.05 were classified as Occasional. Reported variants with MAF ≤0.01 were classified as Rare variants. The variants that were not found in gnomAD were classified as Not reported. Out of the 158 loss-of-function variants identified in the study cohort, 12 variants were Common, eight variants were Occasional, 79 variants were Rare, and 59 variants were Not reported **(A)**. Out of the 6,169 missense variants identified in the study cohort, 751 were Common, 537 were Occasional, 4,160 were Rare, and 721 were Not reported **(B)**. Out of the 16,770 variants with other consequences (including synonymous variants and variants falling in splice regions (20bp flanking exons), intronic regions (outside splice regions), or in the five or three prime untranslated regions of the CVD genes) identified in the study cohort, 3,650 were Common, 1667 were Occasional, 8,656 were Rare, 2,797 were Not reported **(C)**. A bar plot compares the frequency of benign, uncertain significance, and pathogenic variants in families affected by CVD to families with no associated CVD presentations in the Mendelian Study cohort **(D)**. A pie chart visualization of all pathogenic variants that were detected in the Emirati Mendelian Study cohort and not found in both exomes and genomes datasets of the genomAD databases **(E)**.

To evaluate the variants falling in our set of CVD genes in the Emirati Mendelian study cohort, we compared derived minor allele frequencies (MAF) of all retrieved variants filtered from the 125,748 exome sequences available in the Genome Aggregation Database (gnomAD) across different populations, mainly African/African American, Ashkenazi Jewish, Finnish, non-European Finnish, South and East Asian. We grouped all variants based on their functional consequences into Loss-of-Function (LoF), Missense, and Others (Figure 4). We excluded variants of functional unknown implications falling upstream or downstream of CVD genes (Supplementary Table S11).

LoF variants group includes frameshift, splice donor, splice acceptor, initiator codon, stop gained, and stop lost. Of the 158 LoF unique variants detected in this cohort, 79 (62.7%) were found in the gnomAD database. Approximately 50% of the shared LoF variants were of rare allele frequency (MAF ≤0.01; Figure 4A). Notably, 37% (n = 59) of LoF variants identified in the Emirati Mendelian study cohort were missing from the gnomAD exomes datasets (Figure 4A; Supplementary Table S11).

Approximately 88% of our cohort’s 6,169 missense variants (including non-synonymous and in-frame insertion and deletions) were shared with the gnomAD populations. Meanwhile, 67.4% were of rare allele frequency, and 11.7% of variants were missing in the gnomAD exomes (Figure 4B; Supplementary Table S11).

We also investigated other variants found in the Emirati Mendelian Study cohort, such as synonymous variants and variants falling in splice regions (20bp flanking exons), intronic regions (outside splice regions), or in the five or three prime untranslated regions of the CVD genes. Of the 16,770 variants identified in this category, 83.3% were shared with gnomAD populations, 51.6% were of rare allele frequency, and 16.7% were unique to this population (Figure 4C; Supplementary Table S11).

To assess the burden of deleterious variants in patients with CVD from the Emirati Mendelian Study cohort, we classified LoF and missense variants within our CVD gene set into benign, pathogenic, and uncertain significance based on the American College of Medical Genetics and Genomics (ACMG) general variant classification framework. Using Fisher’s exact test, we compared the number of these variants observed in families with CVD presentations (62 variants) to those observed in families with other Mendelian diseases (281 variants). We observed a significant burden of pathogenic variants in the CVD cohort (*p* = 0.0006), while no considerable burden was observed for variants with uncertain significance (*p* = 0.1565) or benign (*p* = 0.9223) consequences (Figure 4D).

To improve power, we repeated the burden analysis on 39 actionable genes, a subset of the 643 CVD genes. We retrieved missense and LoF variants residing in these genes from the study cohort. Out of these variants, we identified the number of pathogenic variants and variants of uncertain significance (Supplementary Figure S11). A significant burden of pathogenic variants was detected in the CVD cohort (*p* = 0.0001). While no significant burden was observed in this set of actionable genes between the CVD cohort and the cohort with other Mendelian diseases (*p* = 0.078) for variants of uncertain significance.

All pathogenic or likely pathogenic variants that were detected in the Emirati Mendelian Study cohort and not found in both exomes and genomes datasets of the genomAD databases are shown in Figure 4E.

### Revealing cardiovascular disease-associated genes and variants from SEHA electronic health records

As genetic screening and testing has become a standard practice in the Genetic Clinic at Tawam Hospital, healthcare providers face challenges interpreting and translating results into clinical care. To determine the nature of test results affecting CVD genes, their interpretation, and their classification of pathogenicity as described in the EHR and to gain insight into the prevalence of associated variants and genes within the UAE population, we examined CVD-specific genes and variants extracted from the EHR for patients who visited Tawam Hospital from 2011 to 2019.

Following a thorough manual examination and curation of 4,860 laboratory reports detailing genetic variations, we identified 693 variants within 269 CVD genes (Supplementary Table S12). All these variants were rare except for 12 benign variants exhibiting a frequency exceeding 0.01 in gnomAD. On the other hand, 21.5% of variants (149/693) were entirely novel, with no representation in public databases like gnomAD and dbSNP or within the clinical literature. The highest count of rare alleles (AC) was observed in genes such as *NF1* (AC = 20), *NAGLU* (AC = 13), *AGL* (AC = 12), *FBXL4* (AC = 12), and *Col1A1* (AC = 10).

**FIGURE 5:**
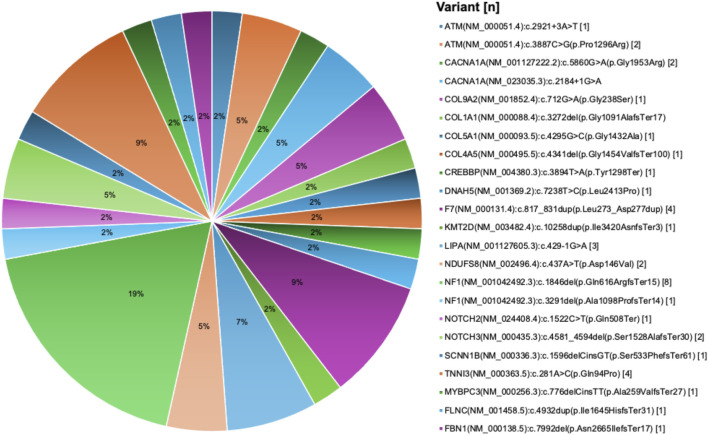
New pathogenic variants in cardiovascular disease genes derived from the UAE SEHA electronic health records. Pie chart visualization of the not reported pathogenic variants showing their frequency in patients records.

Out of the 693 variants, 227 variants were detected in asymptomatic carriers as part of routine genetic screening tests while 466 variants were identified in symptomatic patients. After pathogenicity reassessment following the ACMG classification criteria, 24.8% of variants (116/466) were found to be pathogenic or likely pathogenic (Supplementary Table S12). A significant proportion of the 466 variants, 49.4% (230/466), remained classified as Variants of Uncertain Significance (VUS), while 25.8% (120/466) were categorized as benign or likely benign. Of the 116 pathogenic/likely pathogenic variants identified in patients, 18 variants were new (Figure 5).

Most pathogenic variants were observed as single occurrences in individual patient with some exceptions. For example, the variant *PLOD1* (NM_000302.4):c.955C>T(p.Arg319Ter) was identified in six patients, the *AGL* (NM_000642.3):c.2223_2224del(p.Gln741HisfsTer7) variant was observed in four patients, and both *FBXL4* (NM_012160.4):c.1304G>T(p.Arg435Leu) and *NAGLU* (NM_000263.4):c.1694G>T(p.Arg565Leu) variants were found in three patients each. We also identified eight damaging structural variations in five CVD genes, including deletion encompassing exons 13 to 14 of the *NF1* gene in four patients, deletion of the whole *NF1* gene in one patient, deletion encompassing exons 48 to 52 of *DMD* gene in one patient, duplication encompassing exons 20 to 44 of *DMD* gene in one patient, deletion encompassing exons 51 to 55 of *DMD* gene in two patients, expanded allele of *DMPK* gene in two patients, deletion of exons 20 to 21 of *PCCA* gene in one patients, and whole *FOXC1* gene deletion in one patient.

## Discussion

### Cardiovascular disease genes and pathways

In this work, we first developed an expanded list of genes implicated in heritable CVD and mapped them to their respective pathways. This analysis highlighted the involvement of three critical pathways in most CVD presentations, specifically the Apelin, FoxO, and Ras signaling pathways. These shared pathways might provide a novel idea for further mechanistic studies and hub genes that may serve as novel therapeutic targets for diagnosing and treating inherited and non-inherited forms of CVD. Among these pathways, Apelin signaling assumes prominence as a pivotal player, the sole pathway shared across the five distinct forms of CVD examined in this study. Apelin, recognized as a natural hormone that tends to augment cardiac performance, exhibits a multifaceted tissue expression profile encompassing cardiomyocytes, endothelial cells, and other constituents of the cardiovascular system (Kuba et al., 2019). Its far-reaching influence impacts diverse pathological mechanisms underpinning CVD, including regulating hemodynamic parameters, cardiac contractility, angiogenesis, metabolic equilibrium, cellular proliferation, apoptosis, oxidative stress, inflammatory responses, immune modulation, and pivotal roles in heart development and morphogenesis. Of noteworthy significance, Apelin emerges as a well-established therapeutic target for a spectrum of conditions encompassing hypertension, atherosclerosis, myocardial infarction, and heart failure (Zhong et al., 2017). Furthermore, several studies have detected a noticeable downregulation of Apelin expression in cohorts of patients afflicted with PHD and CM, underscoring the compelling therapeutic potential of targeting this signaling axis to facilitate a diverse array of CVD phenotypes (Kuba et al., 2019; Yang et al., 2022).

FoxO signaling pathway is a complex cascade that revolves around the regulatory activities of the Forkhead box (Fox) family of transcription factors (Zhu, 2016). These factors orchestrate various biological processes, spanning cell cycle dynamics, cellular proliferation, differentiation, migration, metabolic homeostasis, and responses to genomic insults. In the context of CVD, the FoxO signaling pathway assumes a significant role, exerting substantial influence over the development of the heart and actively contributing to an array of pathogenic mechanisms connected to cardiovascular disorders. Such mechanisms encompass aberrant metabolic regulation, heightened oxidative stress, endothelial dysfunction, inflammation, and apoptosis. Previous investigations have elucidated notable aberrations in the expression and function of Fox proteins in patients affected with CHD and CM (Kandula et al., 2016).

Finally, the Ras signaling pathway is known as a principal regulator, overseeing a group of vital signaling networks, including RAF/MEK/ERK/MAPK, PI3K/Akt/mTOR, and Ca++/Calcineurin/NFAT, each stimulating its distinct and consequential signaling cascade (Pan et al., 2007; Roberts and Der, 2007). Ras signaling exerts pleiotropic effects on cell growth and survival with a described role in pathological cardiac remodeling. Genetic perturbations within the Ras signaling pathway and its affiliated cascades underpin an array of cardiac phenotypes with diverse clinical manifestations collectively known as RASopathies, severe forms of CHD such as in Noonan and LEOPARD syndromes. Dysregulated Ras signaling in the heart stimulates cardiomyocyte hypertrophy, usually followed by heart failure (Sugden, 2003). Other studies have shown increased expression of Ras effectors in patients with atrial fibrillation. Therapeutic agents targeting Ras signaling are potential therapies for CVD (Ramos-Kuri et al., 2021).

### Genetics of cardiovascular disease in the United Arab Emirates

Our research explored the genes and variants associated with CVD in a cohort of 3,350 individuals from the UAE, a region notably underrepresented in global genome databases. Despite the diverse ethnic backgrounds of both native and expatriate populations in the UAE (Elliott et al., 2022), our analysis revealed the presence of unique variants specific to this population. These unique variants were identified in the Mendelian cohort (15.5%) and the national EHR dataset (21.5%) compared to populations in the gnomAD database. This discrepancy can largely be attributed to the predominance of European populations in the gnomAD database, a minority within the UAE population.

Despite the disease selection bias in the genetic testing enrolment, clinicians were informed about benign/likely benign variants (29%) and variants of uncertain significance (VUS) (50.6%) within CVD genes, as illustrated in the EHR results. Some variants exceeded gnomAD MAF of 1% (1.7%) or 5% (0.43%). This highlights the uncertainty among diagnostic laboratory experts surrounding whether these variants have any disease-causing potential or are entirely non-impactful, which may lead to confusion among clinicians. No significant association was found when comparing the burden of benign/likely benign and VUS variants in the CVD cohort to the non-CVD cohort within the Mendelian cohort. Therefore, a modified ACMG classification framework should be adapted in the diagnostic labs to determine the clinical significance of sequence variants falling in CVD genes. Indeed, ClinGen’s CM Expert Panel applied a modified ACMG framework to reclassify reported variants in the *MYH7* gene to develop a broadly applicable approach for use in associated disorders aiming to improve consistency for variant interpretation (Rehm et al., 2015; Kelly et al., 2018; Morales et al., 2020). The adapted framework incorporated the complex nature of CVD genetics, including phenotypic, locus, and allelic heterogeneity, reduced and adult-onset penetrance, and the additive effect of multiple variants. Sharing clinical data between diagnostic labs will also aid in improving our ability to classify variants and understand disease development (Kelly et al., 2018).

While the data in this study provides valuable insights, further studies with broader access to comprehensive patient health records, including familial and relatedness information, could enrich the understanding of variant distributions and their implications.

## Data Availability

Data are deposited in the dbSNP database, https://www.ncbi.nlm.nih.gov/SNP/snp_viewTable.cgi?handle=UAEU_GENOMICS_LAB.
