# Supplementary figures and images for: Profiling genetic variants in cardiovascular disease genes among a Heterogeneous cohort of Mendelian conditions patients and electronic health records
Source: Front Mol Biosci. 2024 Oct 1;11:1451457. doi: 10.3389/fmolb.2024.1451457 (PMC11473968; doi:10.3389/fmolb.2024.1451457)

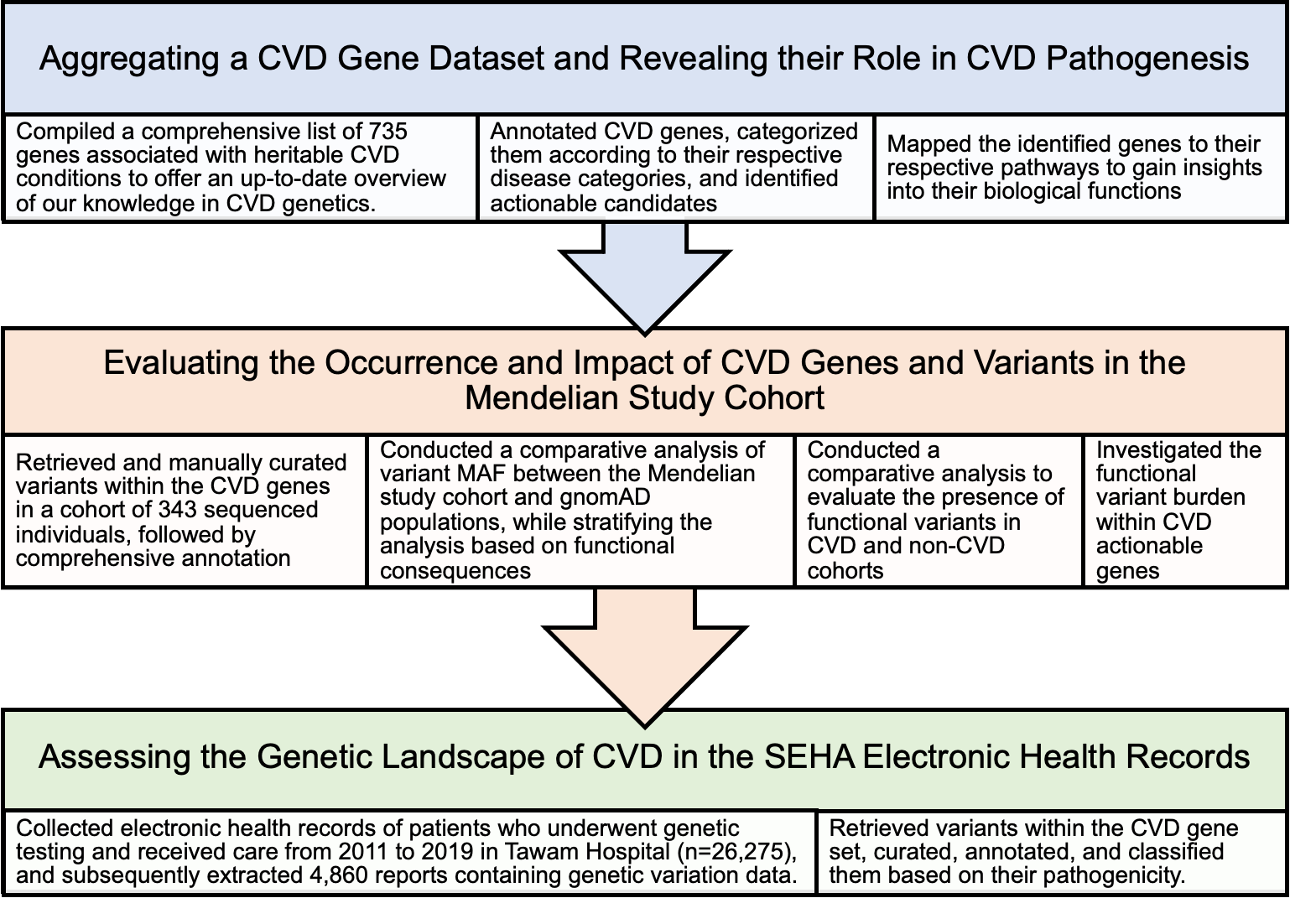

Supplement: Supplementary file 7 [file Image1.PNG]

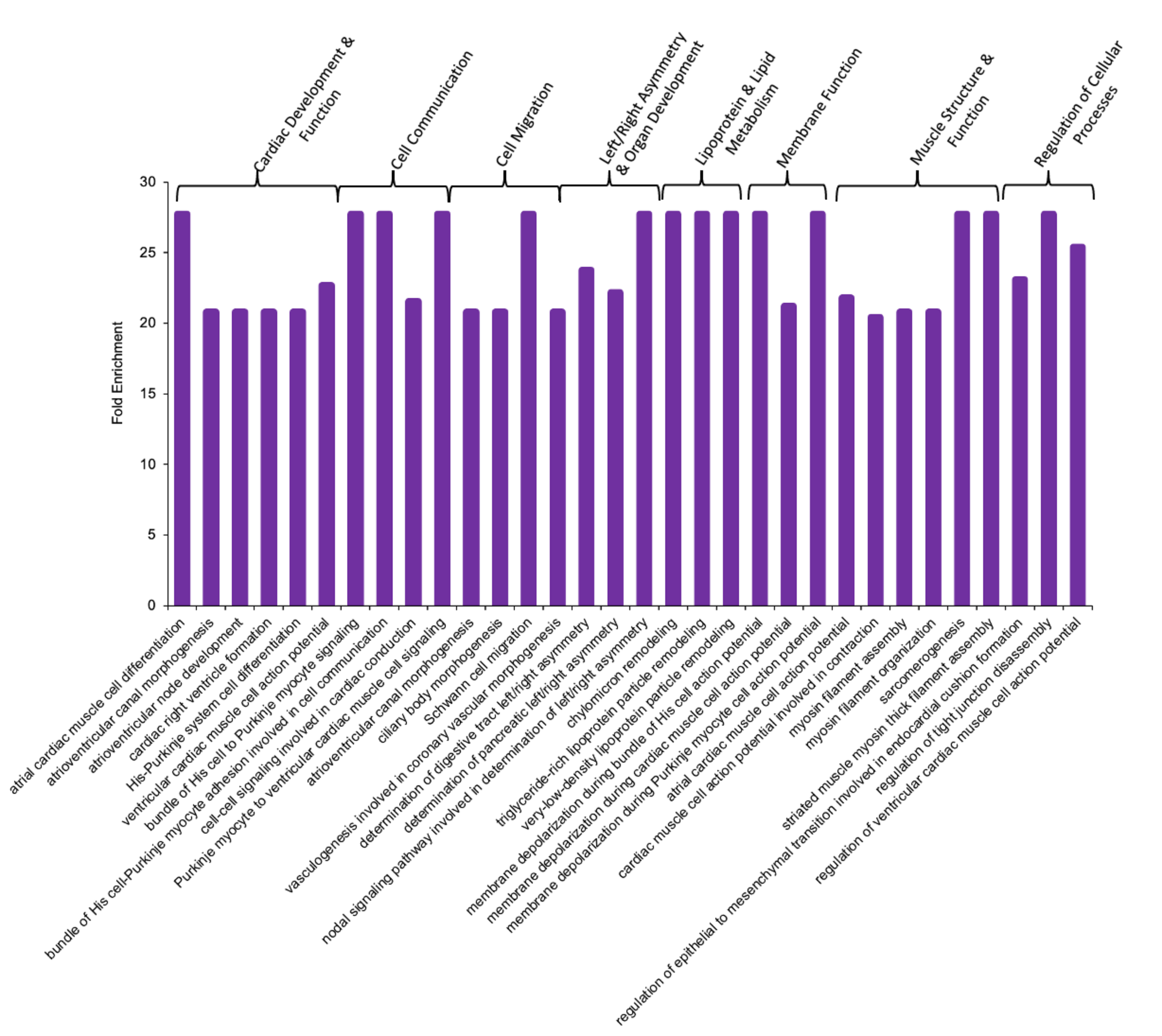

Supplement: Supplementary file 8 [file Image8.PNG]

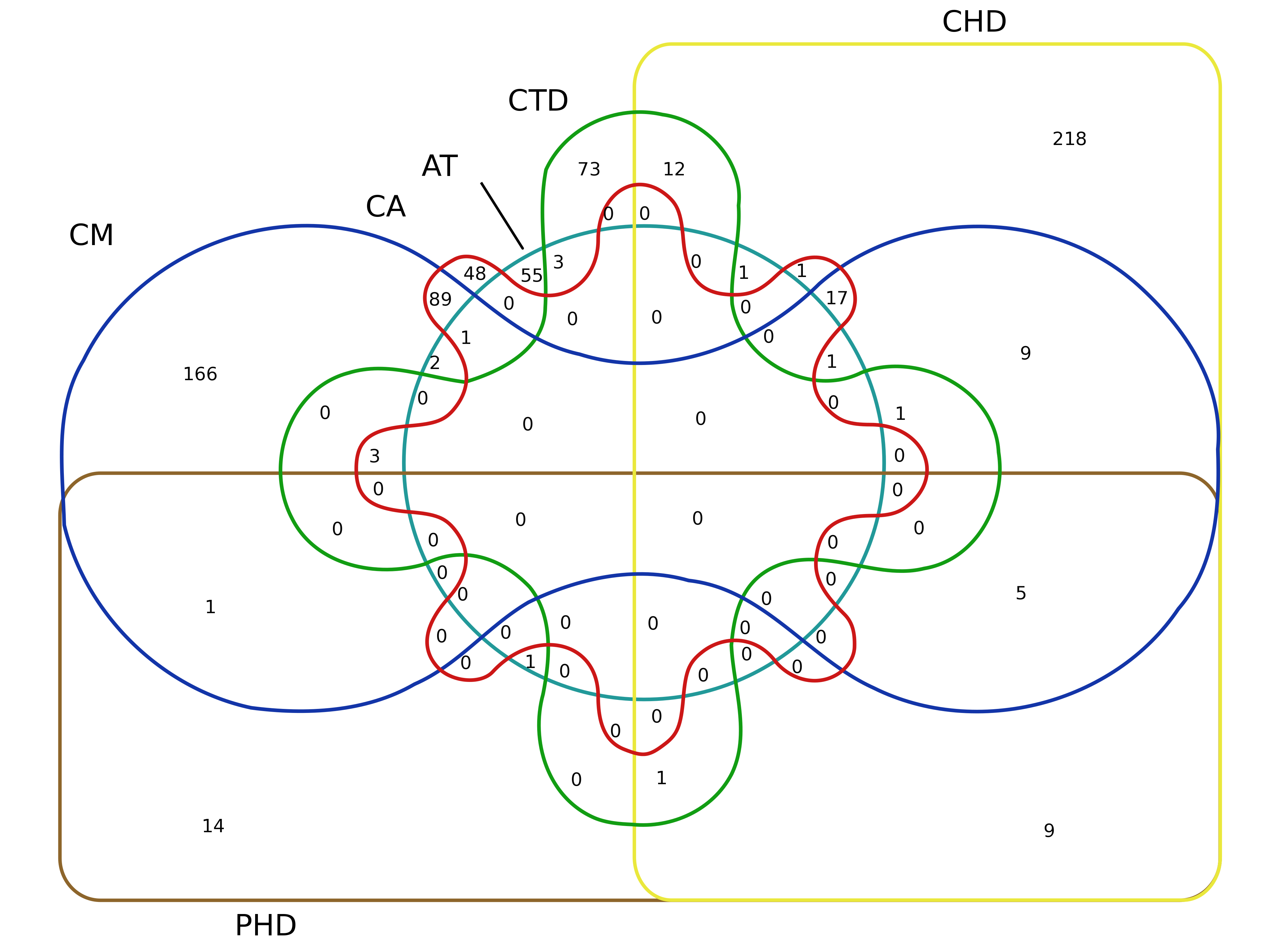

Supplement: Supplementary file 9 [file Image9.PNG]

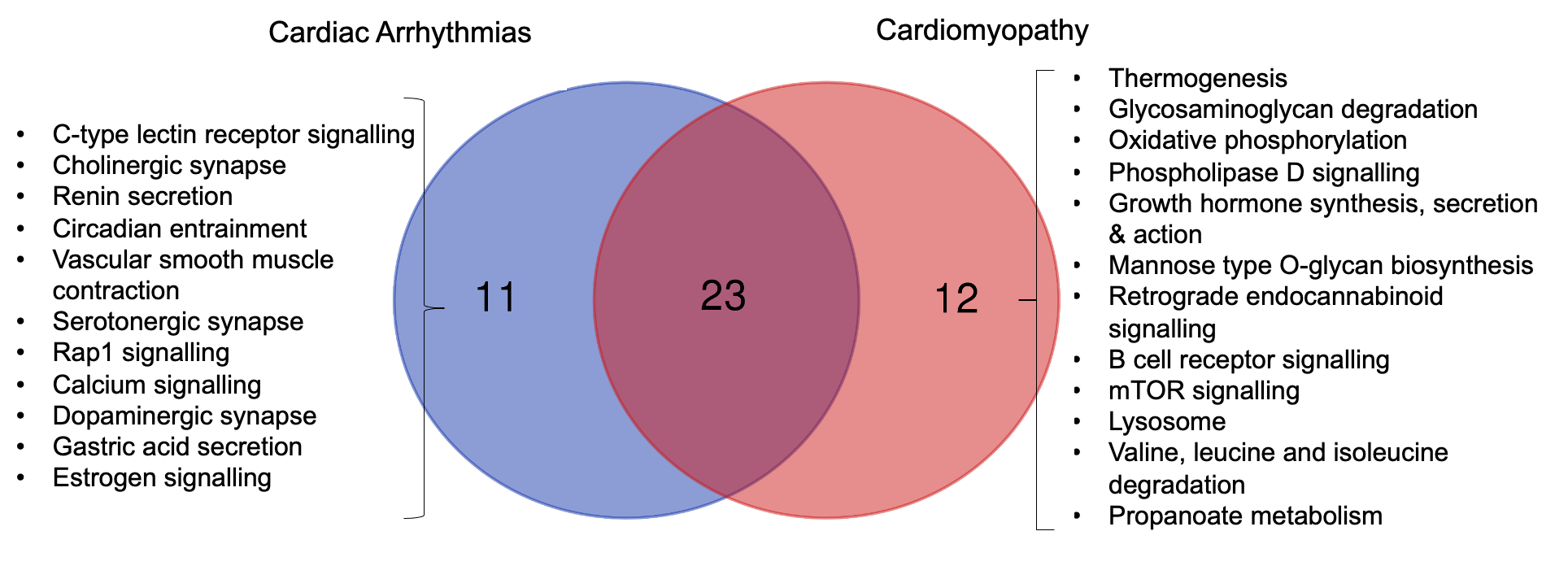

Supplement: Supplementary file 12 [file Image10.PNG]
